# Swine GBP1 restricts PDCoV replication via disrupting the replication and transcription complex formation

**DOI:** 10.1128/jvi.00207-26

**Published:** 2026-04-21

**Authors:** Kunli Zhang, Sutian Wang, Xintong Kang, Fei Li, Ziqiao Zhao, Wuri Nile, Chunhong Zhang, Haiyan Shen, Shouwen Du, Huahua Kang, Mingfei Sun, Zhicheng Liu, Jianfeng Zhang

**Affiliations:** 1 State Key Laboratory of Swine and Poultry Breeding Industry, Guangdong Province Key Laboratory of Livestock Disease Prevention, Key Laboratory for Prevention and Control of Avian Influenza and Other Major Poultry Diseases, Ministry of Agriculture and Rural Affairs, Institute of Animal Health, Guangdong Academy of Agricultural Sciences 117866, Guangzhou, Guangdong, China; 2 Guangdong Provincial Key Laboratory of Animal Breeding and Nutrition, Institute of Animal Science, Guangdong Academy of Agricultural Sciences 117866, Guangzhou, Guangdong, China; University of Kentucky College of Medicine, Lexington, Kentucky, USA

**Keywords:** porcine deltacoronavirus (PDCoV), swine guanylate-binding protein 1 (sGBP1), replication and transcription complex (RTC), non-structural protein 8 (NSP8), antiviral responses

## Abstract

Porcine deltacoronavirus (PDCoV) has been prevalent worldwide for over a decade, causing considerable economic damage to the pig industry and posing a potential threat to public biosecurity. In this study, we found that one of the interferon-stimulated genes (ISGs), swine guanylate-binding proteins 1 (sGBP1), was induced by PDCoV. In the experiments we describe below, we demonstrate that sGBP1 is an antiviral gene inhibiting PDCoV infection by utilizing the sGBP1 stable expression cell line and sGBP1 knockout cell line. Both the large GTPase domain and the α-helical domain are responsible for sGBP1 anti-PDCoV replication. Notably, sGBP1 directly interacts with the scaffold protein NSP8 of the PDCoV replication and transcription complex (RTC). Moreover, the large GTPase domain of sGBP1 interacts with the NTD domain of NSP8, which disrupts the interaction between NSP8 and NSP12 in RTC. In addition, sGBP1 is able to bind with the RNA of PDCoV and inhibits RTC from binding with virus RNA. Here, our research uncovers a new mechanism through which sGBP1 inhibits PDCoV replication. This finding not only deepens our comprehension of the antiviral roles of ISG molecules but also offers a promising target for the prevention of PDCoV infections.

**IMPORTANCE:**

PDCoV is an enteric coronavirus that has garnered significant global attention due to its current prevalence in causing diarrhea and even mortality in pigs, as well as its broad host range encompassing poultry, rodents, ruminants, and even humans. Understanding the mechanism by which host factors inhibit viral replication is critical for developing effective antiviral strategies. Here, we found that PDCoV induced swine guanylate-binding proteins 1 (sGBP1) to inhibit viral replication. Our study first reveals that sGBP1 impairs the replication and transcription complex (RTC) formation through two ways: (i) competes with the RNA-dependent RNA polymerase (RdRp) NSP12 to bind with NSP8; (ii) sGBP1 binds with PDCoV RNA to inhibit the RNA binding of RTC. Our results uncover a previously unknown antiviral mechanism of GBP1, offering a promising target for the prevention of viral infections.

## INTRODUCTION

Porcine deltacoronavirus (PDCoV), an enteric coronavirus, has been extensively circulating worldwide for more than 10 years, causing newborn piglets and sows with acute diarrhea, vomiting, dehydration, and even death (1–3). This virus belongs to the *Coronaviridae* family, the genus *Deltacoronavirus*, and is characterized as an enveloped, single-stranded RNA virus possessing a positive-sense orientation (4). Unlike other swine enteric coronaviruses, PDCoV could infect multiple species, including pigs, bovines, chickens, mice, and humans (5–10). In 2021, plasma specimens obtained from three Haitian children presenting with acute undifferentiated febrile illness were confirmed to be positive for PDCoV strains (10). This discovery suggests that PDCoV might already have the capacity to transmit between different species. However, knowledge on PDCoV infection, pathogenesis, disease, and prevention remains limited.

The genome of PDCoV measures approximately 25.4 kb and encodes four structural proteins—spike (S), membrane (M), envelope (E), and nucleocapsid (N)—in addition to 3 accessory proteins (NS6, NS7, NS7a) and 15 non-structural proteins, designated NSP2 through NSP16 (11, 12). Coronavirus genome replication is mediated by the replication and transcription complex (RTC) (12, 13). In severe acute respiratory syndrome coronavirus 2 (SARS-CoV-2), the RTC comprises NSP7, NSP8, NSP9, NSP12, and NSP13 (14). The minimal essential core for coronavirus RNA synthesis is formed by the NSP12–NSP7–NSP8 complex (15). The RTC has been considered one of the most promising targets for controlling coronavirus replication. The core component of this complex is the catalytic subunit (NSP12) of an RNA-dependent RNA polymerase (RdRp) (15). Multiple studies indicate that NSP8 functions as a crucial scaffold, providing the platform for NSP7 and NSP12 binding, and facilitating the attachment of NSP9, NSP13, and RNA to the SARS-CoV-2 RTC (16–19). Moreover, the viral NSP8 is indispensable for maintaining the stability of the RTC and ensuring the replication of the coronavirus.

Virus infection and replication are tightly regulated by the innate immune response. Once the virus invades, type I and type III IFNs rapidly activate local responses to control viral spread, followed by the activation of type II IFN, which orchestrates a stronger, antigen-specific immune response. During the process, over 2000 interferon-stimulated genes (ISGs) are induced by Type-I, Type-II, or Type-III IFNs through Janus kinase-signal transducer and activator of transcription (JAK-STAT) signaling pathways to inhibit viral replication at different stages of the viral lifecycle (20, 21). The guanylate-binding proteins (GBPs) are one type of ISGs, with hydrolase activity, which helps to hydrolyze GTP into GDP and GMP (22, 23). The importance of GBPs in host defense to protozoan pathogens, bacterial, and viral has been known for some time. Of these, GBP1 has been subject to the most extensive research. Human GBP1 (hGBP1) is known to confer protection against Japanese encephalitis virus (JEV), Hepatitis E virus (HEV), vesicular stomatitis virus (VSV), encephalomyocarditis virus (EMCV), Kaposi’s sarcoma-associated herpesvirus (KSHV), influenza A virus (IAV), and SARS-CoV-2 (24–29). The antiviral mechanism of GBP1 varies depending on the type of virus. The antiviral effect of GBP1 depends on its GTPase function in HCV, CSFV, and VSV infection (27, 30–32). But in HEV infection, the antiviral effect of GBP1 depends on its capacity to homodimerize (33). HGBP1 impedes nuclear export of KSHV virions by impairing the actin filament formation (28). Chicken GBP1 (chGBP1) inhibits Infectious Bronchitis Virus (IBV) infection through degrading IBV-N protein in the autophagy pathway (34). The antiviral capacity of GBPs has been proven to be species-specific. In contrast to human data, overexpression and knockout mouse GBP1 (mGBP1), even mGBP1/2/3/5, show no antiviral activity against IAV, HSV-1, SeV, LCMV, and EMCV *in vitro* and *in vivo* (35). A recent report reveals that hGBP1 inhibits the ancestral SARS-CoV-2 strain and dominant variants of concern (VOCs) (29). Its antiviral activity does not depend on the mechanisms observed in GBP2 and GBP5, such as actin remodeling and interfering with furin-mediated S protein cleavage (29, 36). To date, the specific mechanism by which GBP1 combats mammalian coronavirus infection remains unclear.

Here, we found that PDCoV infection induces the expression of swine GBP1 (sGBP1), which inhibits PDCoV replication. Mechanistically, sGBP1 interacts with PDCoV NSP8 to disrupt the NSP8 and NSP12 interaction. Moreover, the sGBP1 binds with PDCoV RNA and reduces the RNA-binding content of the RTC. The sGBP1 disrupts the formation of RTC in these two ways to inhibit the replication of PDCoV. Our research uncovered a previously unrecognized antiviral function of sGBP1, offering a promising target for coronavirus prevention and control.

## RESULTS

### SGBP1 is induced by PDCoV

**Fig 1:**
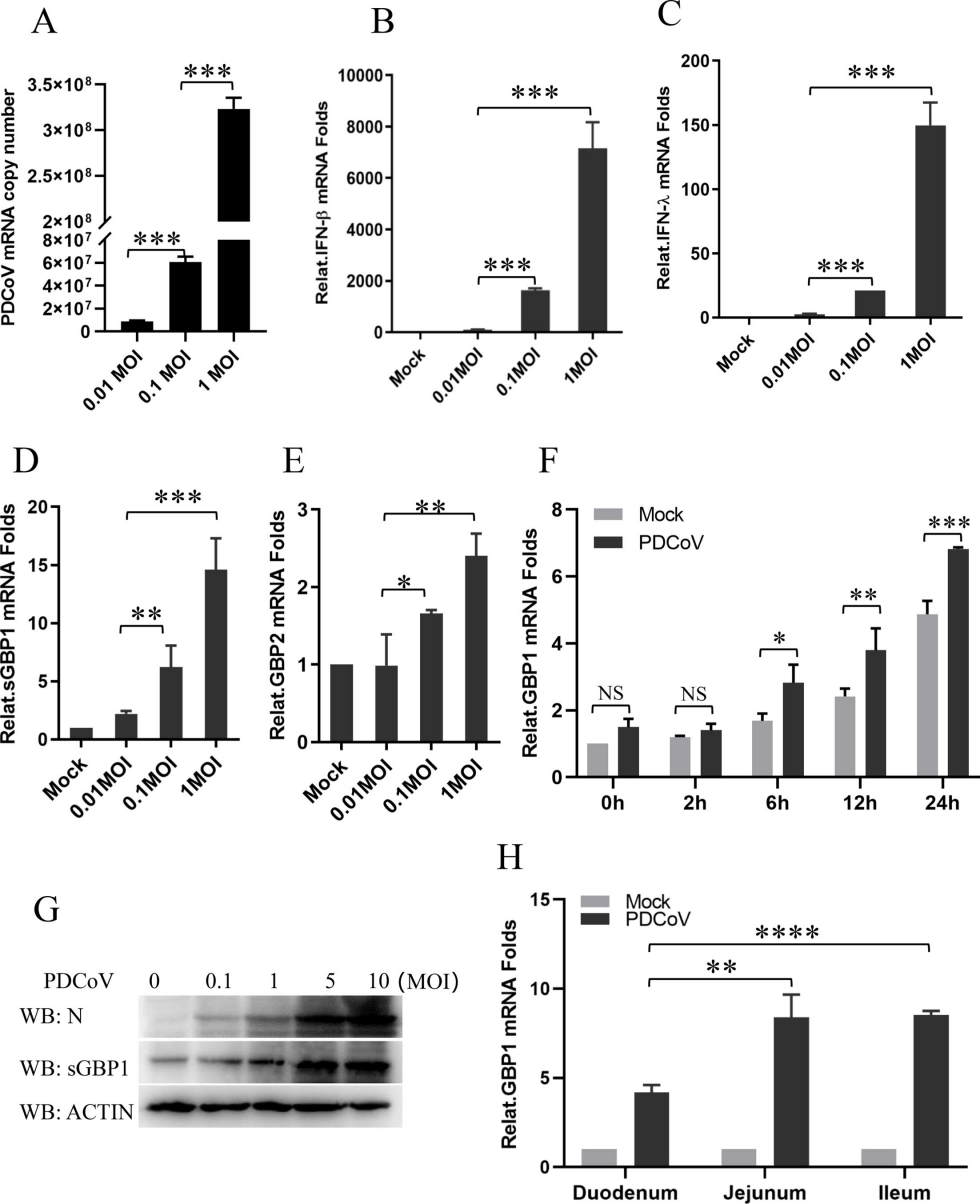
PDCoV infection regulates the sGBP1 gene expression. Cells were exposed to viruses at an MOI of 0.01, 0.1, or 1 for 12 h. Absolute quantification of PDCoV genome copies was performed through quantitative PCR (qPCR) (**A**). Additionally, quantitative PCR was employed to measure the relative expression levels of IFNβ (**B**), IFNλ (**C**), sGBP1 (**D**), and sGBP2 (**E**) in the infected cells. (**F**) Cells were either mock-infected or infected at 0.1 MOI for 0, 2, 6, and 12 h. Cells were collected at these specified intervals for total RNA extraction, and sGBP1 mRNA levels were assessed by qPCR. (**G**) Infection of cells with PDCoV at 0.1, 1, 5, and 10 MOI for 12 h, followed by western blot analysis to detect the protein expression of PDCoV N protein and GBP1. (**H**) The relative expression of sGbp1 mRNA in intestinal tissues from PDCoV-infected piglets was determined through qPCR analysis. NS, not significant; *P < 0.05, **P < 0.01, ***P < 0.001, and ****P < 0.0001.

GBPs are important immune defense factors against viral infection (37–39). Two swine GBPs (sGBP1 and sGBP2) have been reported to be related to porcine virus infection (28, 40, 41). To assess whether sGBP1 or sGBP2 takes part in PDCoV infection, the ST cells were infected with 0.01, 0.1, and 1 MOI of PDCoV for 12 h. The PDCoV copy number and the mRNA levels of IFNβ, IFNλ, sGBP1, and sGBP2 were confirmed by absolute and relative quantification or quantitative real-time PCR (qPCR) analysis. The ST cells successfully established the PDCoV infection, and the IFNβ and IFNλ were significantly induced by PDCoV (Fig. 1A through C). At the same time, the levels of mRNA for the interferon downstream ISG genes sGBP1 and sGBP2 were increased in a dose-dependent manner after stimulation for 12 h (Fig. 1D and E). But the increase folds of sGBP1 are higher than those in sGBP2. Hence, we focus on sGBP1 in the following study. The dynamic relative sGBP1’s mRNA expression folds were analyzed after PDCoV (0.01 MOI) infected 0 h to 24 h (Fig. 1F). From PDCoV infected 6 h to 24 h, the sGBP1’s mRNA expression was significantly increased compared with mock-infected cells. Additionally, we tested the impact of PDCoV infection on sGBP1 protein expression through a Western blot assay. The result is consistent with mRNA transcription (Fig. 1G). The main target organs of PDCoV infection in piglets are the jejunum and ileum segments of the small intestine (42). The relative expression of sGBP1 mRNA in small intestinal tissues from PDCoV-infected piglets was detected through qPCR analysis. The results showed that the mRNA level of sGBP1 was significantly enhanced, especially in the jejunum and ileum segments (Fig. 1H). All results showed PDCoV could induce sGBP1 expression.

### Overexpression of sGBP1 inhibited PDCoV replication

**Fig 2:**
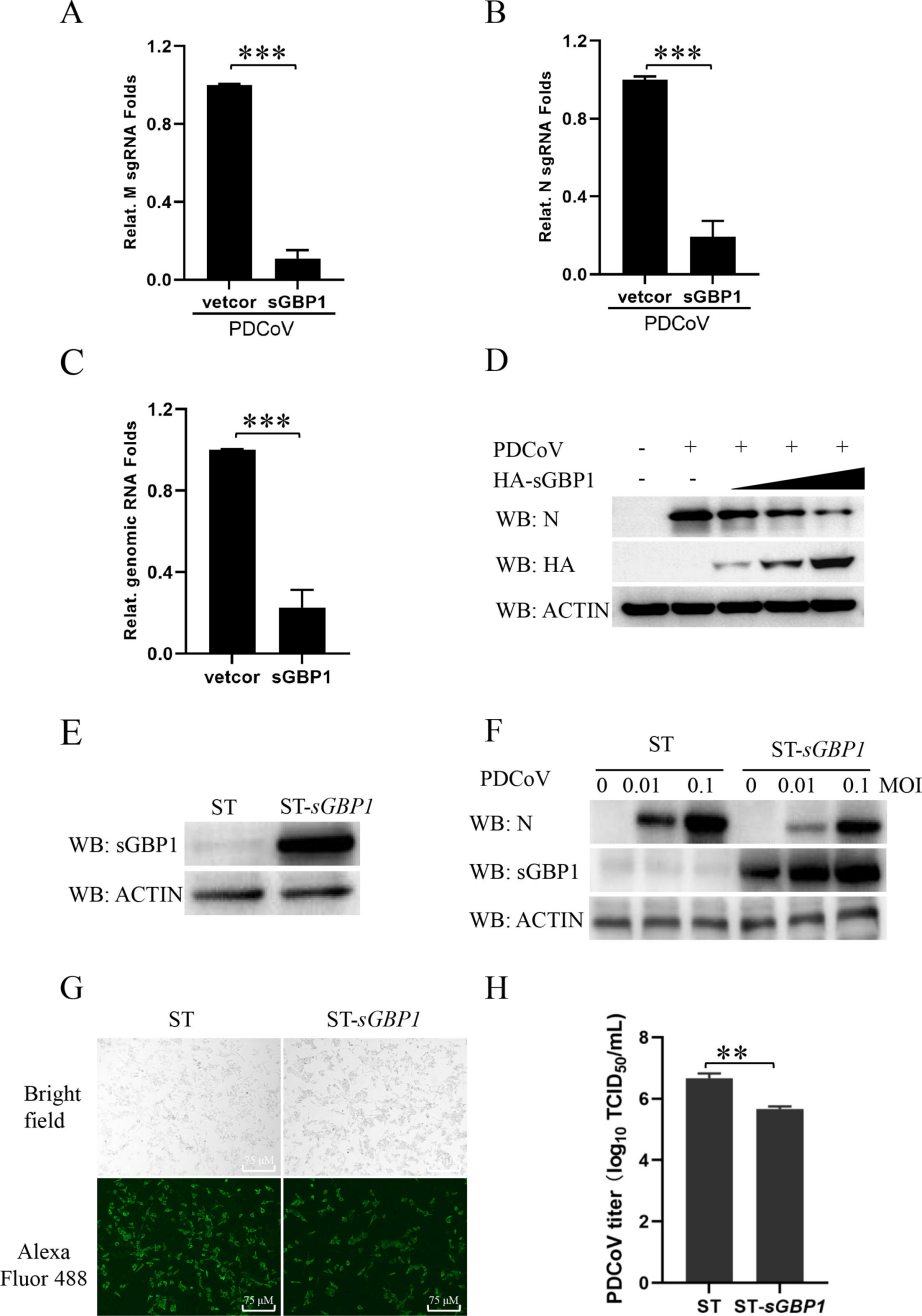
sGBP1 is a negative regulator of PDCoV replication. (**A–C**) ST cells were transfected with either pCAGGS-HA or pCAGGS-HA-sGBP1. After 24 h post-transfection, these cells were infected with PDCoV at 0.1 MOI for 12 h. At 36 h post-transfection, cells were harvested, and the total RNA was extracted. The M (**A**), N (**B**) sgRNA, and genomic RNA (**C**) were detected to quantify PDCoV replication by qPCR. (**D**) ST cells received transfections of pCAGGS or pCAGGS-HA-sGBP1 at doses of 0.5, 1, or 2 μg. Following 24 h post-transfection, cells were infected with PDCoV and then lysed for protein extraction. Western blotting was used to assess the N protein and HA-tagged protein levels. (**E**) ST cells stable overexpressing sGBP1 were generated via a lentiviral system. The expression of sGBP1 in both ST and ST-*sGBP1* cell lines was detected using western blot analysis. (**F**) Both ST and ST-*sGBP1* cell lines were infected with 0, 0.01, and 0.1 MOI PDCoV. After 12 h post-infection, cells were collected, and viral replication was evaluated by Western blot. (**G and H**) ST and ST-*sGBP1* cells were infected with PDCoV at 0 or 0.1 MOI. Cells were then fixed using 4% paraformaldehyde, and PDCoV N protein was confirmed by immunostaining, visualized as green fluorescence; scale bar represents 75 µm (**G**). Additionally, cells were harvested to determine viral titers through TCID_50_ assays (**H**). **P < 0.01,***P < 0.001.

To detect the role of sGBP1 during PDCoV infection, we transfected the HA tag sGBP1 plasmid (HA-sGBP1) into cells and infected the cells with 0.1 MOI PDCoV for 12 h. Quantitative PCR was used to measure the PDCoV copy number. Fig. 2A through C shows that the overexpression of sGBP1 could significantly decrease PDCoV M, N genes’ sgRNA, and genomic RNA transcription levels. Western blot analysis consistently showed that elevated sGBP1 expression downregulated the PDCoV N protein (Fig. 2D). To confirm the inhibiting function of sGBP1 on PDCoV replication, an ST cell line stably overexpressing sGBP1 protein was established by lentiviral, and the cell line was named ST-*sGbp1*. The sGBP1 expression in ST wild-type cells and ST-*sGbp1 cells* was measured by western blotting (Fig. 2E). Subsequently, the ST wild-type cells and ST-*sGbp1* cells were treated with PDCoV for 12 h. Fig. 2F showed few PDCoV N proteins were detected in ST-sGbp1 cells with an MOI of 0.01 and 0.1. Immunofluorescence assay (IFA), alongside the median tissue culture infective dose (TCID_50_) assay, was used to evaluate PDCoV replication level. As visualized in Fig. 2G, the fluorescence intensity of the N protein in ST-*sGbp1* cells is notably lower compared to that observed in ST wild-type cells. Consistent with the results of Western blot and IFA, the viral titer of PDCoV was markedly decreased by the stable overexpression of sGBP1 (Fig. 2H). Hence, the overexpression of sGBP1 inhibits the PDCoV replication.

### Knockout of sGBP1 promotes PDCoV replication

**Fig 3:**
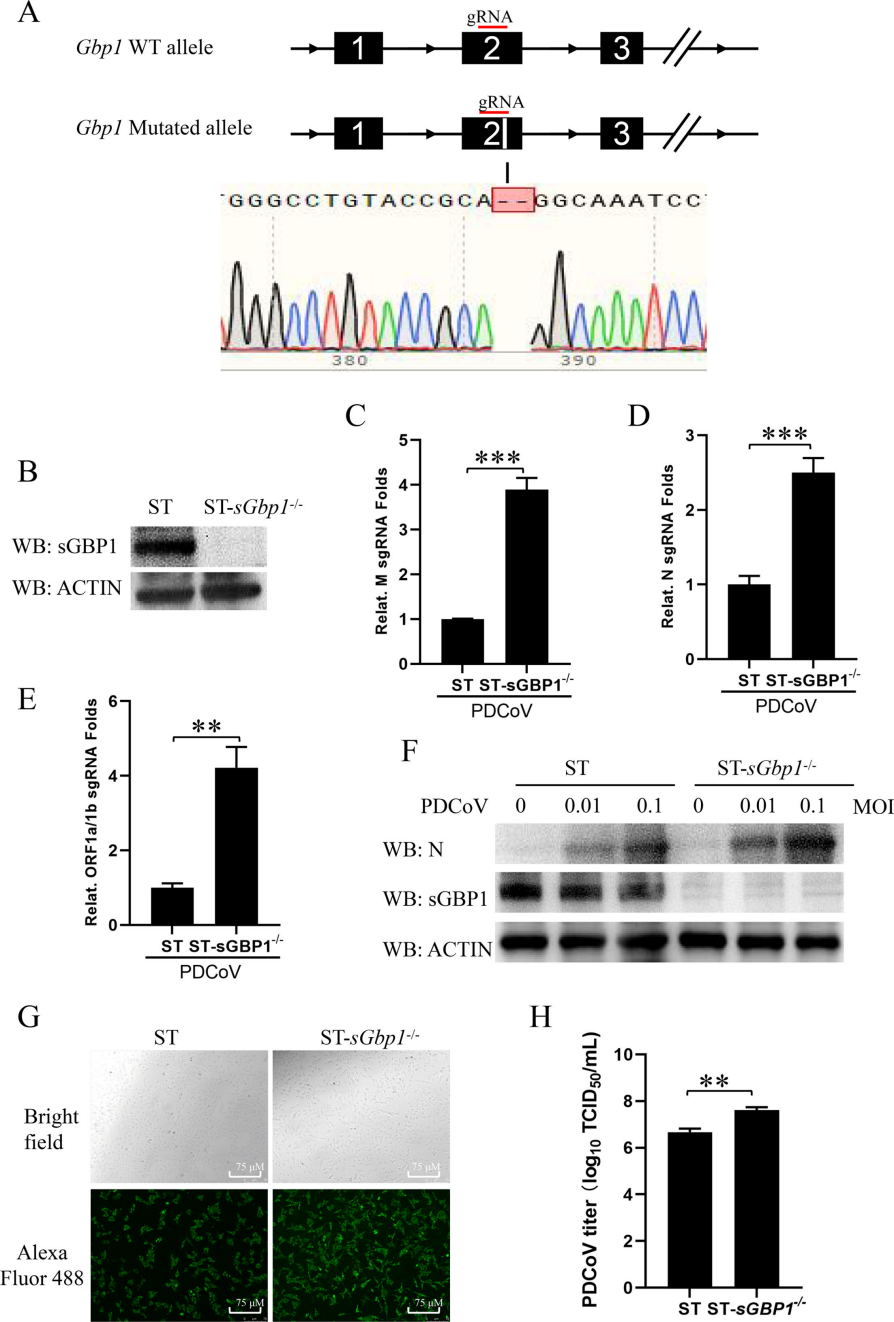
knockout sGbp1 promotes PDCoV replication. The ST cells gene was edited with *sGbp1*-specific target *sGbp1* by the CRISPR/Cas9 system. The sGbp1 knockout ST cell line was named ST-*sGbp1*^−/−^. (**A**) Schematic diagram of CRISPR/Cas9-mediated *sGbp1* gene knockout ST cell line. The sgRNA target sites were marked in a red line. The sGbp1 gene of the ST-*sGbp1*^−/−^ cell line was sequenced, and the deficient bases were marked in a red frame. (**B**) Western blot analysis was performed to assess sGBP1 protein levels in both ST-sGbp1 knockout (ST-*sGbp1*^−/−^) and wild-type ST cells. (**C–E**) The ST and ST-*sGbp*1^−/−^ cell lines were infected with PDCoV at MOIs of 0.1. After 12 h post-infection, cells were harvested for total RNA extraction, and quantitative RT-PCR was used to measure the sgRNA expression of the PDCoV M (**C**), N (**D**), and genomic RNA (**E**). (**F**) The ST and ST-*sGbp1*^−/−^ cell lines were infected with PDCoV at MOIs of 0.01 and 0.1, respectively. After 12 h post-infection, western blotting was conducted to detect the protein levels of PDCoV N, sGBP1, and ACTIN. (G and H) Both ST and ST-*sGbp1*^−/−^ cells were infected with 0.1 MOI PDCoV for 12 h. IFA was applied to evaluate the expression of the N protein, with a scale bar indicating 75 μm (**G**). Subsequently, cells were collected to test viral titers via TCID_50_ assays (**H**). **P < 0.01,***P < 0.001.

To confirm the antiviral function of sGBP1 in PDCoV infection, the CRISPR/Cas9 technology was employed to construct the *sGbp1* gene knockout cell line. The sGbp1 gene in ST cells was subject to a deletion of two bases in exon 2, which was achieved using a special gRNA (Fig. 3A). Due to the deletion of the two bases, a frameshift mutation occurred in the *sGbp1* gene from the 49th amino acid. The western blot results have shown that the sGBP1 protein is absent in ST-sGbp^−/−^ cells (Fig. 3B). To clarify the effect of knocking out sGBP1 on PDCoV replication, we infected ST wild-type cells and ST-*sGbp^−/−^* cells at 0.1 MOI of viruses, respectively. Viral replication levels were evaluated at 12 h post-infection through QPCR. The absence of sGBP1 markedly enhanced PDCoV replication in M, N sgRNA, and genomic RNA (Fig. 3C through E). Consistent with the virus RNA results, the N protein expression levels were significantly improved in ST-*sGbp^−/−^* cells compared with ST wild-type cells (Fig. 3F). The visual assessment of the impact of knocking out sGBP1 on PDCoV replication was conducted through IFA to detect N protein expression levels in both ST wild-type cells and ST-*sGbp^−/−^* cells. Compared to the findings in ST-*sGbp1* cells, ST-*sGbp^−/−^* cells showed a substantial rise in the green fluorescence intensity of the N protein following PDCoV (0.1 MOI) infection (Fig. 3G). After infecting ST wild-type cells and ST-*sGbp^−/−^* cells with PDCoV at 0.1 MOI for 12 h the TCID_50_ assay was employed to quantify the viral titer. The viral titer of PDCoV in ST-*sGbp^−/−^* cells was significantly increased compared with ST wild-type cells (Fig. 3H). According to the above results, we found sGBP1 is a key host gene to inhibit PDCoV replication.

### The suppression of PDCoV replication by sGBP1 is mainly due to its intrinsic GTPase function

**Fig 4:**
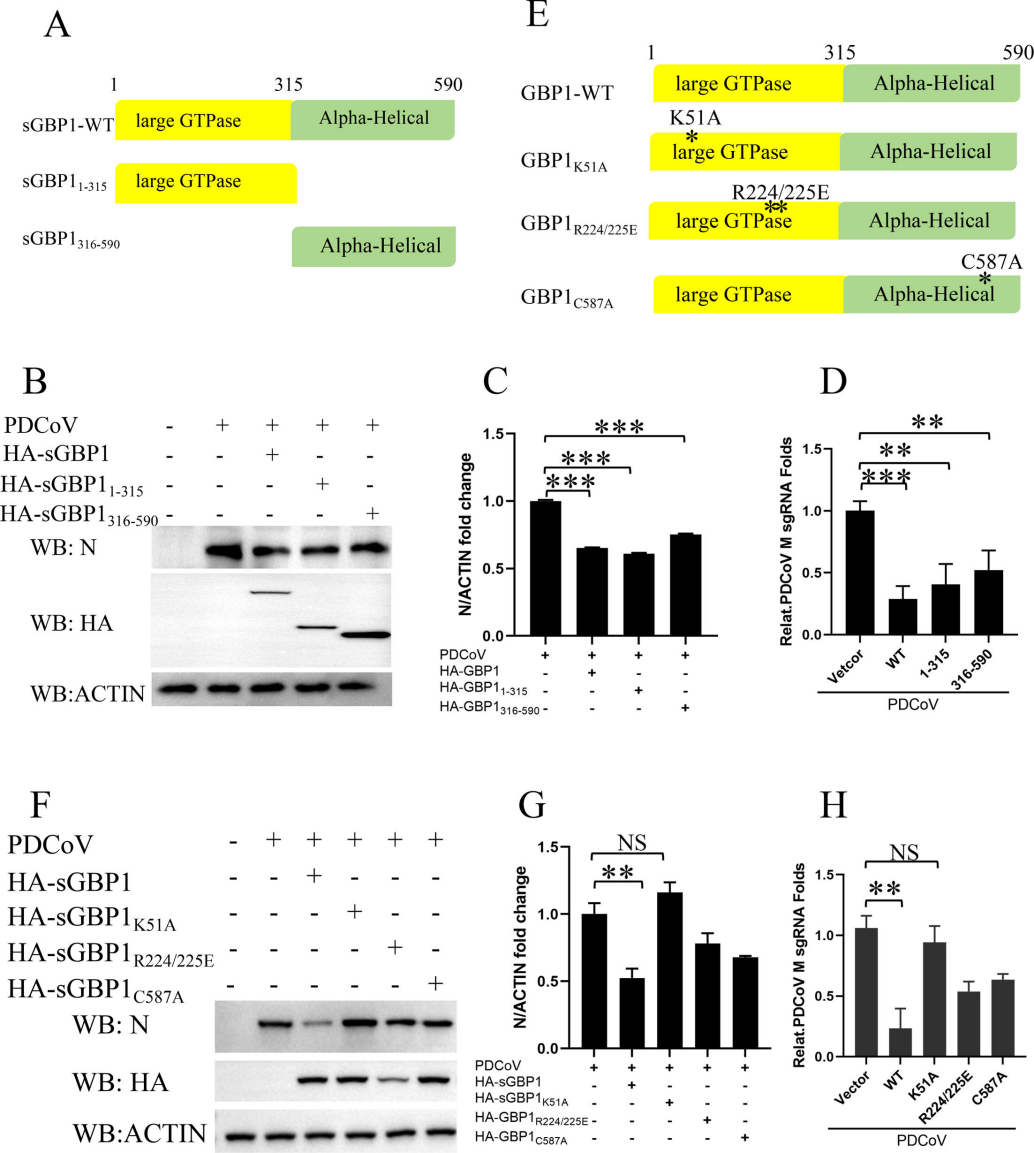
The GTPase activity is mainly responsible for sGBP1 anti-PDCoV replication. (**A**) Schematic diagram of sGBP1. (**B–D**) After 24 h post-transfection (plasmids encoding pCAGGS-HA, pCAGGS-HA-sGBP1, pCAGGS-HA-sGBP1_1-315_, and HA-sGBP1_316-590_), the cells were infected with PDCoV at an MOI of 0.1 for 12 h. Following infection, cells and culture supernatants were collected separately. And the PDCoV N protein expression was assessed by western blotting (**B**). The relative abundance of N protein in the groups expressing pCAGGS-HA-sGBP1, pCAGGS-HA-sGBP1_1-315_, HA-sGBP1_316-590_, and the empty vector control was quantified and normalized to ACTIN levels using ImageJ software (**C**). Viral nucleic acids were extracted from infected cells, and viral load was measured by quantitative PCR (qPCR) (**D**). (**E**) A schematic representation of the sGBP1 mutant constructs is provided. (**F–H**) ST cells were transfected with plasmids encoding pCAGGS-HA, pCAGGS-HA-sGBP1, and mutants pCAGGS-HA-sGBP1_K51A_, HA-sGBP1_R224/225E_, and HA-sGBP1_C587A_ for 24 h. Subsequently, cells were infected with PDCoV at an MOI of 0.1 for 12 h. Western blot was used to evaluate N protein levels (**F**), which were normalized to ACTIN using ImageJ (**G**). Additionally, qPCR was conducted to determine the related PDCoV mRNA change fold within the cells (**H**). NS, not significant; *P < 0.05, **P < 0.01, and ***P < 0.001.

GBP1 is a protein with multiple domains, featuring a sizable GTPase domain at the N-terminus and an α-helical segment located at its C-terminus (43, 44). To explore the functional domains of sGBP1 during PDCoV infection, we generated truncated plasmids that individually express the large GTPase domain and the α-helical region (Fig. 4A). After overexpressing the full-length plasmid and the truncated plasmids in ST cells, PDCoV with an infection dose of 0.1 MOI was introduced. The N protein and M sgRNA transcription levels were then detected, respectively. The anti-PDCoV activity of sGBP1 is mediated by both its large GTPase domain and α-helical domain (Fig. 4B through D). In order to further explore the active site of sGBP1, we constructed three point-mutated plasmids of sGBP1 (Fig. 4E). The K51 is a key site for the execution of the GTPase-cycle function in GBP1. Mutation K51A abolished the GTPase activity of GBP1 (45, 46). The self-assembly and nucleotide hydrolysis of hGBP1 are facilitated by the two amino acid residues Arg227 and Lys228, and mutation of the R227/K228 to E made hGBP1 with constitutive homodimerization (47). A comparison of the sequences indicates that hGBP1 R227/K228 corresponds to swine GBP1 R224/K225. The C587A mutant triggers sGBP1-lacking isoprenylation (45, 48, 49). Unlike the wild-type protein, the expression of the K51A mutant showed a marginal change in the PDCoV-N protein and RNA (Fig. 4F through H). The results indicated that the antiviral effect of sGBP1 against PDCoV is dependent on its GTPase activity. Whereas the expression of the R224/K225E mutant group did not show stronger antiviral function on PDCoV replication compared to the sGBP1 wild-type group. Indicating that the constitutive homodimerization of sGBP1 is not crucial for the suppression of the PDCoV proliferation. At the same time, the antiviral levels of C587A were lower than the sGBP1 wild-type plasmid group. Collectively, these findings confirm that the GTPase function is vital for sGBP1 to effectively suppress PDCoV replication, with the isoprenylation processes of sGBP1 also contributing to this inhibitory mechanism.

### SGBP1 interacts with NSP8 of PDCoV

**Fig 5:**
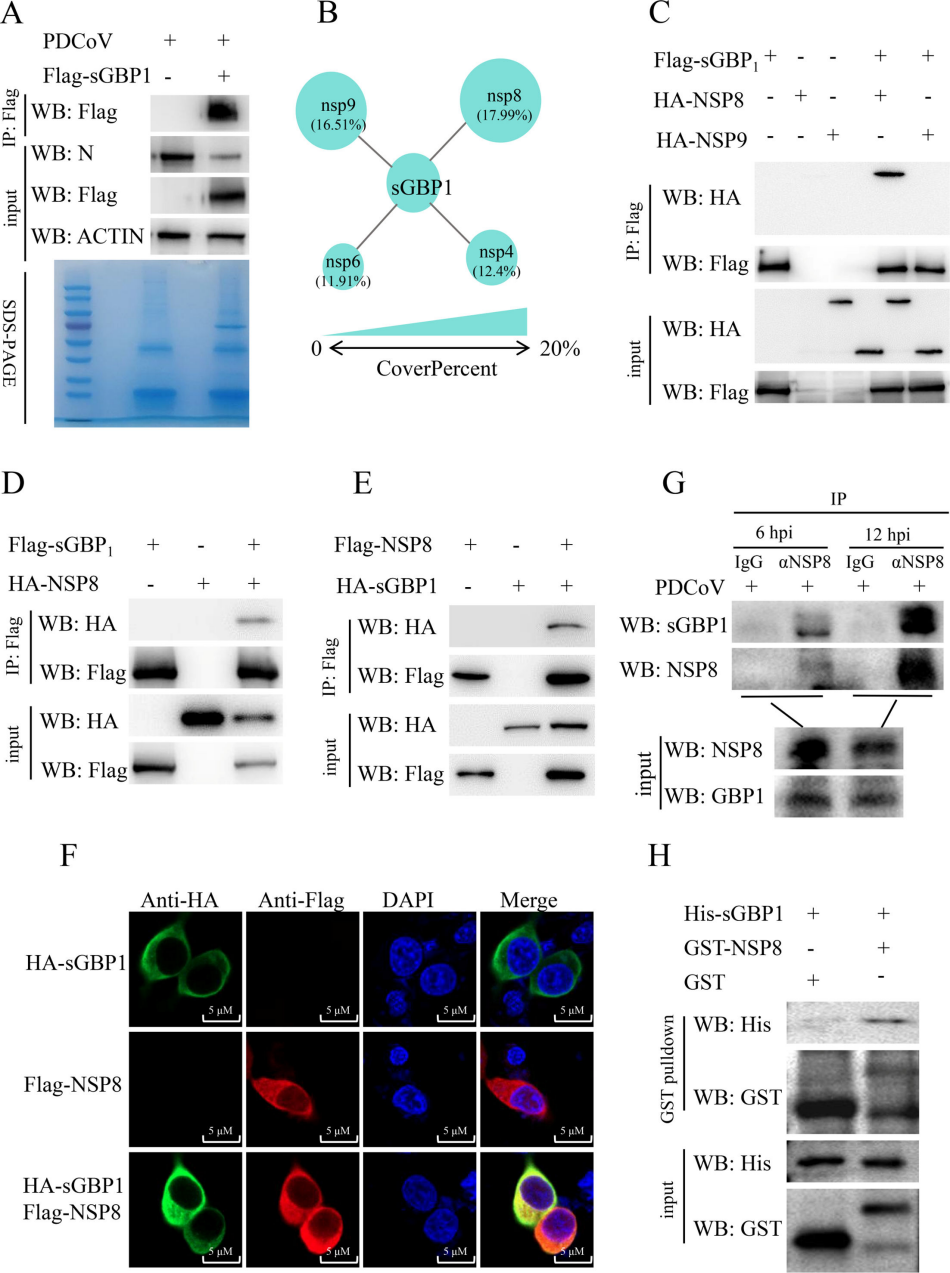
sGBP1 directly interacts with PDCoV NSP8. Cells were transfected with either the mock or the pCAGGS-FLAG-sGBP1 plasmid. After 24 h post-transfection, both groups were infected with 0.1 MOI PDCoV for 12 h. (**A**) Cell lysates were subjected to Co-IP utilizing an anti-FLAG antibody. Subsequently, FLAG-sGBP1, along with its interacting proteins, was eluted using IP washing buffer. Western blot assay was conducted on both the immunoprecipitated complexes and portions of whole-cell lysates using anti-FLAG, anti-N, and anti-ACTIN antibodies. The immunoprecipitated samples were resolved using SDS-PAGE and subsequently stained with Coomassie Brilliant Blue; two specific gel lanes were then excised for further mass spectrometry (MS) analysis. (**B**) Proteins interacting with sGBP1 were identified through MS, with circles of varying sizes representing coverage percentages. (**C**) HEK-293T cells were transfected with a FLAG-sGBP1 plasmid alongside either a control or HA-NSP8, or HA-NSP9 plasmid. At 24 h post-transfection, cells were harvested and subjected to Co-IP. Western blotting was performed on both whole-cell lysates and immunoprecipitates. (**D**) HEK-293T cells were co-transfected with pCAGGS-FLAG-sGBP1 and either pCAGGS-HA-NSP8 or mock vector plasmids. After 24 h, cells were collected for Co-IP and western blot. (**E**) Similarly, HEK-293T cells were co-transfected with pCAGGS-FLAG-NSP8 and either pCAGGS-HA-sGBP1 or mock vector plasmids for Co-IP. (**F**) HEK293T cells were transfected with FLAG-NSP8, HA-sGBP1, or both. Green: rabbit anti-HA monoclonal antibody; Red: mouse anti-FLAG monoclonal antibody; Blue: nuclei. Scale bar: 5 μm. (**G**) Cells infected with PDCoV (0.1 MOI) were collected at 6 and 12 h for endogenous Co-IP, and anti-NSP8 antibody was used in the Co-IP. IgG served as a negative control. (**H**) Purified GST fusion proteins of PDCoV NSP8 or GST alone were incubated with purified His-tagged sGBP1. Western blot analysis of the proteins of His-sGBP1 and GST-NSP8 in the GST beads.

To identify the PDCoV proteins that interact with sGBP1, the co-immunoprecipitation (Co-IP) and mass spectrum (MS) were performed. The MS data showed that in the FLAG-sGBP1 group, NSP6, NSP4, NSP9, and NSP8 coverage percent increased above 10% compared with the FLAG-empty plasmid group (Fig. 5A and B). We selected NSP9 (16.51%), NSP8 (17.99%), which with the highest coverage, to verify their interactions with sGBP1. The Co-IP result showed that only HA tag NSP8 could interact with FLAG-sGBP1 (Fig. 5C). Moreover, exchanging the tags of NSP8 and sGBP1 and then conducting the Co-IP experiment again. Whether it is the sGBP1 of the flag tag or the sGBP1 of the HA tag, the sGBP1 interacts with NSP8 in HEK293T cells (Fig. 5D and E). The subcellular co-location of HA-sGBP1 and FLAG-NSP8 proteins was examined in HEK293T cells. The result demonstrated the co-location of sGBP1 and PDCoV NSP8 protein in the cytoplasm (Fig. 5F). Consistent with these results, endogenous sGBP1 interacts with PDCoV NSP8 protein in ST cells (Fig. 5G). The GST pull-down assay was conducted to investigate a potential direct physical association between sGBP1 and PDCoV NSP8. As illustrated in Fig. 5H, His-sGBP1 was efficiently precipitated by GST-NSP8 but not by GST alone. Collectively, these findings indicate that sGBP1 directly binds to NSP8.

### The extensive GTPase region of sGBP1 binds to the NTD of NSP8

**Fig 6:**
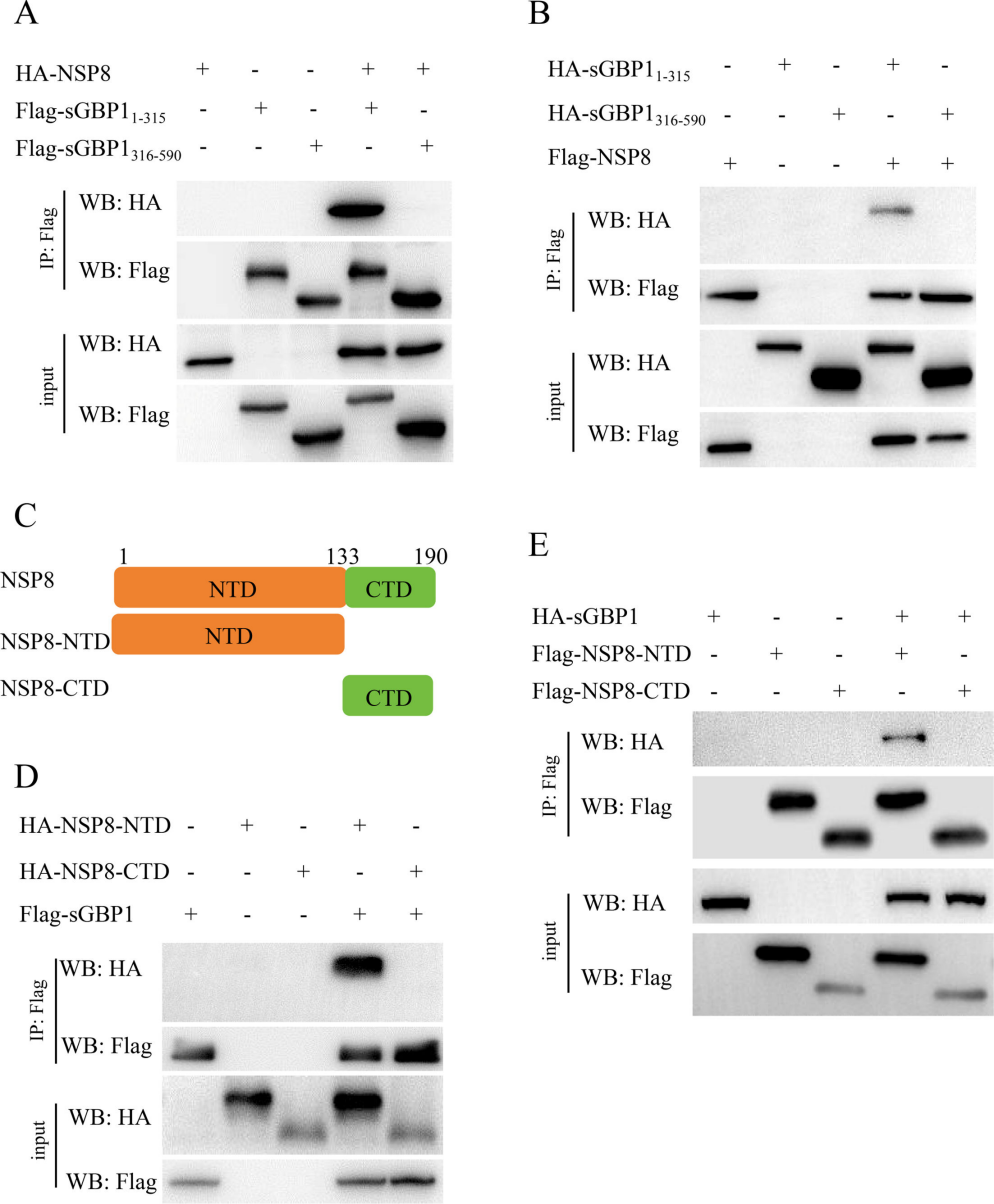
The GTPase domain of sGBP1 interacts with the NSP8 NTD. (**A**) HEK-293T cells were transfected with pCAGGS-FLAG-sGBP1_1-315_, FLAG-sGBP1_316-590_, and either pCAGGS-HA-NSP8 or the pCAGGS-HA vector control. After 24 h post-transfection, cells were collected for Co-IP. (**B**) HEK-293T cells were co-transfected with pCAGGS-FLAG-NSP8, along with pCAGGS-HA-sGBP1_1-315_, pCAGGS-HA-sGBP1_316-590_, or the HA vector plasmid. Cells were harvested at 24 h post-transfection for Co-IP. (**C**) A schematic diagram of NSP8. (**D**) HEK-293T cells were co-transfected with pCAGGS-FLAG-sGBP1 and either pCAGGS-HA-NSP8-NTD, pCAGGS-HA-NSP8-CTD, or the HA vector plasmid. After 24 h, cells were harvested for Co-IP. (**E**) HEK-293T cells were co-transfected with plasmids encoding pCAGGS-FLAG-NSP8-NTD, pCAGGS-FLAG-NSP8-CTD, pCAGGS-HA-sGBP1, or the HA vector control. At 24 h post-transfection, cells were collected and subjected to Co-IP.

Our findings reveal that the N-terminal large GTPase domain, together with the C-terminal helical domain of sGBP1, both contribute to antiviral activity against PDCoV (Fig. 4). In order to map the interaction domain of sGBP1 and PDCoV NSP8, HEK293T cells were simultaneously transfected with FLAG-tagged truncated variants of sGBP1 (sGBP1_1-315_, sGBP1_316-590_) and HA-tagged NSP8. Results from the Co-IP assay indicate that the large GTPase domain of sGBP1 associates with PDCoV NSP8 (Fig. 6A). To confirm the result, HA tag plasmid of sGBP1-large GTPase domain and sGBP1-helical domain were conducted and co-transfected with FLAG-NSP8 to HEK293T cells (Fig. 6B). Consistent with Fig. 6A, sGBP1’s large GTPase domain is responsible for PDCoV NSP8 interaction (Fig. 6B).

The NSP8 of PDCoV consists of 190 amino acids. It is classified into NTD (1–133 aa) and CTD (134–190 aa) (Fig. 6C). FLAG and HA tags NSP8 plasmids were employed to ensure the interaction domain of NSP8 and sGBP1. The results showed that the only NTD domain interacts with sGBP1 (Fig. 6D and E). To sum up, we confirmed that the sGBP1 GTPase domain engages with the NSP8 at its N-terminal domain. These data suggest that sGBP1 exerts its antiviral activity against PDCoV by targeting the NTD of NSP8.

### SGBP1 suppresses PDCoV replication by interfering with the binding between NSP8 and NSP12

**Fig 7:**
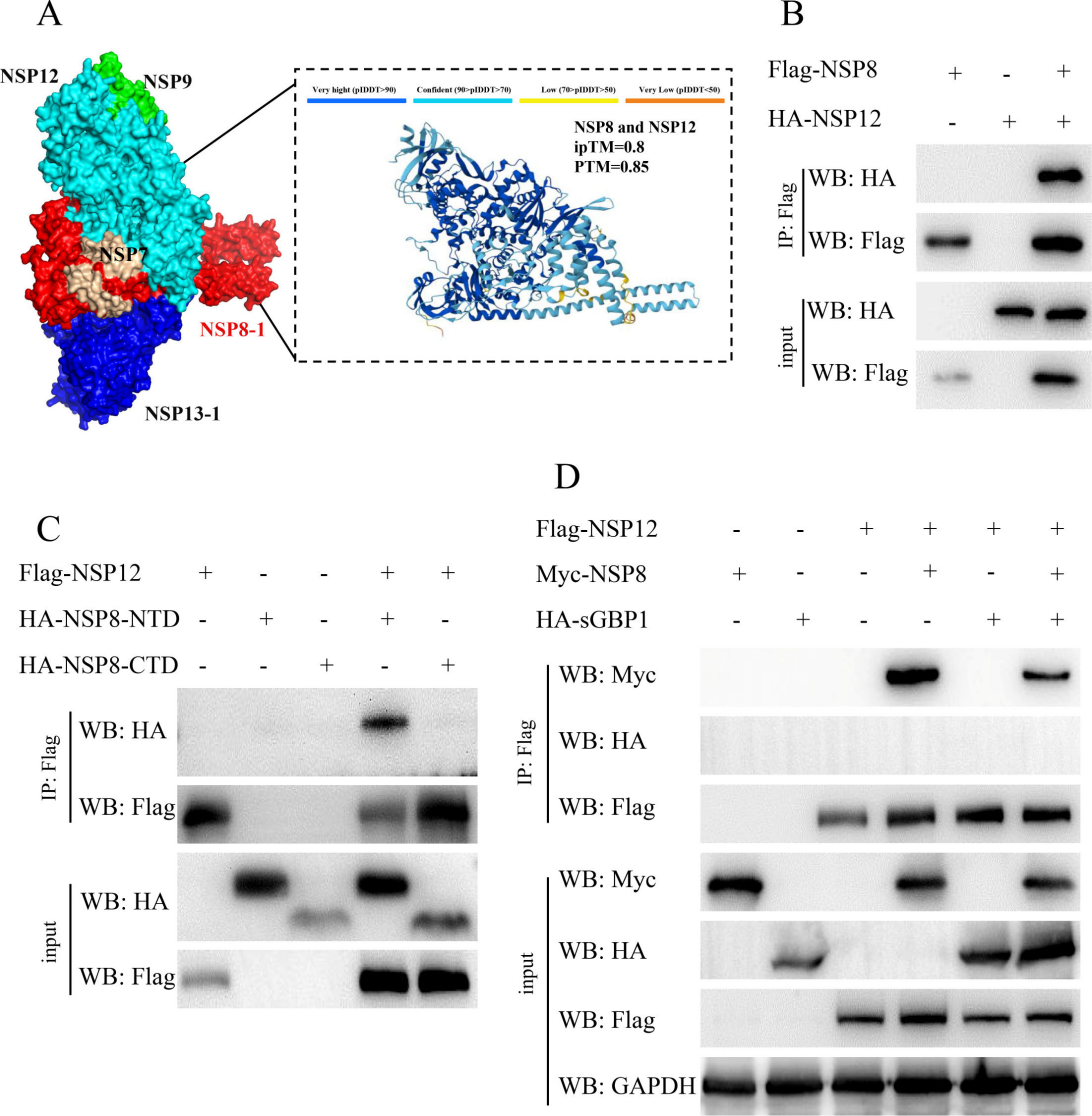
SGBP1 disrupts the interaction between NSP8 and NSP12. (**A**) The structural model of the PDCoV RTC complex and the molecular docking model of NSP8-NSP12 were predicted and evaluated through AlphaFold 3. pTM + ipTM ≥ 0.5: the proteins have the possibility of interacting; pTM + ipTM ≥ 0.75: The possibility of protein interaction is high. The ipTM score and pTM score of the model represent the confidence of the prediction. (**B**) HEK-293T cells were co-transfected with pCAGGS-FLAG-NSP8, pCAGGS-HA-NSP12, or the pCAGGS-HA vector control. After 24 h post-transfection, cells were collected for Co-IP. (**C**) HEK-293T cells were co-transfected with pCAGGS-FLAG-NSP12 along with pCAGGS-HA-NSP8-NTD, pCAGGS-HA-NSP8-CTD, or the HA vector plasmid. Cells were harvested at 24 h post-transfection for Co-IP. (**D**) HEK-293T cells received co-transfections of pCAGGS-FLAG-NSP12, pCAGGS-Myc-NSP8, and either pCAGGS-HA-sGBP1 or the HA vector control. After 24 h, cells were collected for Co-IP.

NSP8 and NSP9 represent essential elements within the RTC of coronaviruses (14, 17). In the IP-MS results, in the overexpression of the sGBP1 group, the cover percent of NSP8 and NSP9 was higher than other proteins of PDCoV, suggesting that sGBP1 might be involved in the RTC formation of PDCoV (Fig. 4A). Studies have established that NSP12, RNA-dependent RNA polymerase (RdRp), serves as the central component of the RTC. During the replication cycle of coronavirus, NSP12 is anchored by viral RNA along with NSP7, NSP8, NSP9, and NSP13 proteins to assemble the RTC (14). To assess the binding of PDCoV NSP8 and NSP12 within the RTC complex, AlphaFold 3 was employed to predict the structural model of the PDCoV RTC complex, along with molecular docking models for NSP8 and NSP12. The RTC structure model of PDCoV indicates that NSP8 and NSP12 form a tight cluster, which is similar to SARS-CoV-2. Both the iPTM score (0.8) and PTM score (0.85) for the NSP8-NSP12 interaction fall within the high confidence interval, indicating that the predicted complex structure closely matches the actual structure. This result strongly suggests a high probability of robust interaction between the two proteins (Fig. 7A). Consequently, we detected the interaction between PDCoV NSP8 and NSP12 within HEK293T cells. Consistent with our expectation, the NSP8 and NSP12 of PDCoV interact with each other (Fig. 7B). The interaction domain between NSP8 and NSP12 has also been tested as follows. As Fig. 7C shows, NSP8 combines with NSP12 through its NTD domain. These results have revealed that the N-terminal domain of NSP8 is involved in the interaction process between it and sGBP1 (Fig. 6C and D), as well as NSP12 (Fig. 7B and C). We speculated that the interaction between sGBP1 and NSP8 might disrupt the formation of PDCoV RTC to inhibit virus complications. To verify our hypothesis, these plasmids FLAG-NSP12, Myc-NSP8, and HA-sGBP1 were transfected into HEK293T cells either individually or in combination for 36 h. The competitive Co-IP results show that there is no interaction between sGBP1 and NSP12. However, once sGBP1 is added, the interaction between NSP12 and NSP8 is weakened (Fig. 7D). The above results reveal that sGBP1 and NSP8 interaction interferes with the association between NSP8 and NSP12 of RTC, ultimately suppressing viral transcription and replication processes.

### SGBP1 disrupts PDCoV genomic RNA binding with the replication and transcription complex

**Fig 8:**
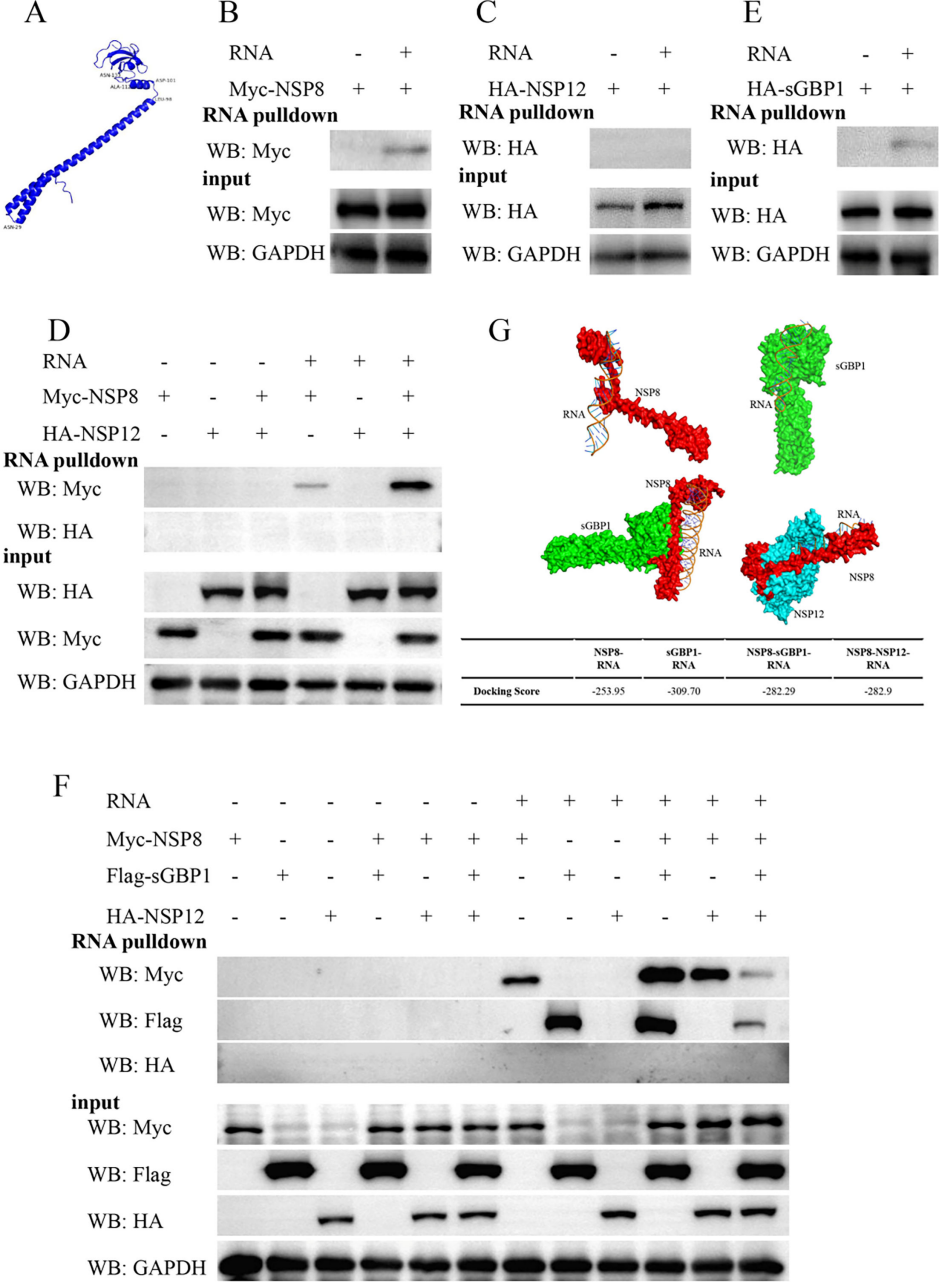
SGBP1 inhibits PDCoV RNA binding to the replication and transcription complex (RTC). (**A**) Predicted the PDCoV NSP8 structure model by the AlphaFold 3 server website. (**B**) HEK293T cells were transfected with MYC-NSP8 for 24 h. Subsequently, the cell lysates were incubated with 20 μg of either unlabeled or biotinylated PDCoV RNA for 6 h. The resulting biotinylated RNA-protein complexes were captured and analyzed through Western blot. (**C**) HEK293T cells were transfected with HA-NSP12. After 24 h post-transfection, cells were collected for RNA pull-down assays. (**D**) Lysates from HEK293T cells expressing MYC-NSP8, HA-NSP12, or both proteins were separately incubated with 20 μg of unlabeled or biotinylated PDCoV RNA for RNA pull-down assays. (**E**) HEK293T cells were either mock-transfected or transfected with the HA-sGBP1 for 24 h. Cell lysates were collected for RNA pull-down assays. (**F**) HEK293T cell lysates expressing MYC-NSP8, HA-NSP12, FLAG-sGBP1, or both proteins were individually incubated with 20 μg of unlabeled or biotinylated PDCoV RNA. The complexes, after precipitation using streptavidin beads, were subsequently analyzed through Western blot assay. (**G**) The AlphaFold 3 and HDOCK were employed to predict the binding ability of NSP8, sGBP1, sGBP1-NSP8, and NSP8-NSP12 to PDCoV dsRNA. The Docking Score indicates the binding affinity between the protein complex structure and the RNA. It is inversely correlated with predicted binding stability and affinity; a more negative value signifies stronger binding.

Predicted the PDCoV NSP8 structure model was processed in the AlphaFold 3 online server website and PYMOL software for visualization (Fig. 8A). Our analysis revealed that PDCoV NSP8 NTD autonomously folds into a helix-turn-helix motif followed by a disordered segment, resembling the structural features observed in SARS-CoV-2 NSP8. Functioning as a crucial anchoring scaffold within the viral RTC, NSP8 plays a core role in RNA binding (17). Hence, we collected the cell lysate of HEK293T cells that contained ectopically expressed PDCoV NSP8 protein and used the RNA pull-down assay to analyze whether NSP8 could bind to the photosensitive biotin-labeled PDCoV RNA. Figure 8B shows that NSP8 binds with PDCoV RNA. In addition, we also detected the RNA-binding activity of NSP12. However, isolated ectopic expression of NSP12 cannot bind to the RNA of PDCoV (Fig. 8C). Since NSP8 interacts with NSP12, combined with other NSPs to form RTC initiate the transcription and replication of the coronavirus (14, 50). We hypothesize that NSP12 might affect the binding ability of NSP8 to the viral RNA. As Fig. 8D shows that when NSP8 and NSP12 are co-expressed, the binding PDCoV RNA ability of NSP8 is enhanced, but NSP12 still does not bind with PDCoV RNA. We have proved that sGBP1 interacts with NSP8 and inhibits the interaction between NSP8 and NSP12 (Fig. 7). We speculated that sGBP1 might also affect the binding of NSP8 to RNA. To assess the RNA-binding capability of sGBP1, an RNA pull-down assay was conducted. Interestingly, sGBP1 was able to bind with the RNA of PDCoV (Fig. 8E). Our further research found that the interaction between NSP8 and sGBP1 significantly enhanced their RNA binding ability (Fig. 8F). Moreover, once sGBP1, NSP8, and NSP12 were expressed together, the binding amount of NSP8 to RNA significantly decreased. In order to explain this phenomenon, we used AlphaFold 3 and HDOCK to predict the binding ability of NSP8, sGBP, NSP8s-sGBP1, and NSP8-NSP12 to PDCoV RNA. Molecular docking results showed that the docking score for the NSP8s-sGBP1 complex with PDCoV RNA is −282.29, which means its binding affinity is higher than that for NSP8 alone with RNA (−253.95). The docking scores for the NSP8-sGBP1-RNA (−282.29) and NSP8-NSP12-RNA (−282.9) are similar. Furthermore, sGBP1 exhibits higher affinity for RNA than NSP8 (Fig. 8G). It means that the addition of sGBP1 competes with the NSP8-NSP12 complex binding with PDCoV RNA. In summary, the association between sGBP1 and NSP8 not only interferes with the NSP8-NSP12 interaction but also competes with the RTC complex for binding to PDCoV RNA, ultimately leading to the inhibition of PDCoV replication.

## DISCUSSION

**Fig 9:**
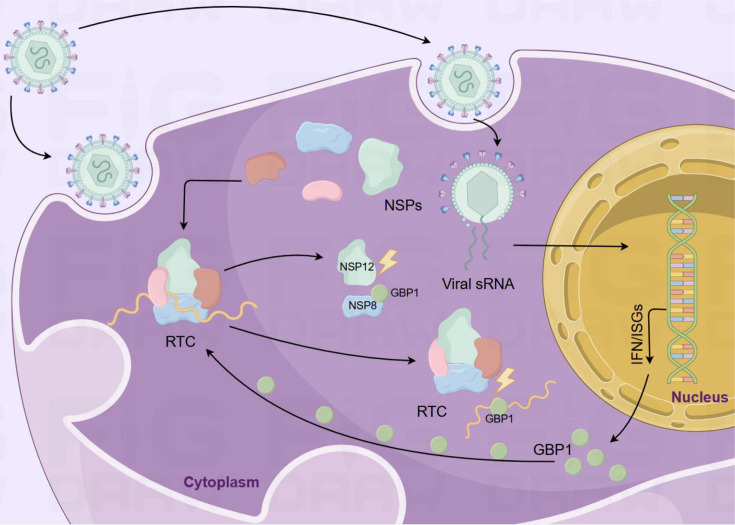
The antiviral mechanism of sGBP1 inhibits PDCoV replication. PDCoV infection induced sGBP1 expression. Mechanistic diagram showing that sGBP1 impairs the replication and transcription complex (RTC) formation through two ways: sGBP1 impairs the replication and transcription complex (RTC) formation through two ways: (i) competes with the RNA-dependent RNA polymerase (RdRp) NSP12 to bind with NSP8; (ii) sGBP1 binds with PDCoV RNA to inhibit the RNA binding of RTC.

PDCoV, a recently identified member of the coronavirus family, has drawn growing concern because of its significant impact on the global swine industry’s economy and its possible implications for human health (2, 10, 51). Understanding the mechanisms underlying host factors that fight against viral replication is critical for developing effective antiviral strategies. Although some recent studies have reported the ability of the host’s GBP1 to combat coronavirus infections, the specific mechanism by which it works remains unclear (29, 34). Our findings reveal for the first time a novel regulatory mechanism by which sGBP1 counteracts PDCoV replication. In this research, we discovered that PDCoV triggers the expression of sGBP1, which functions as an antiviral host factor during infection. The GTPase function of sGBP1 serves as a key factor in inhibiting PDCoV replication. The large GTPase domain of sGBP1 interacts with NSP8, a key component of the RTC, and disrupts its association with NSP12. Moreover, sGBP1 impairs the RNA-binding capacity of the RTC complex, leading to suppressed viral replication (Fig. 9). Given the effective anti-coronavirus function of the GBP family proteins (29, 36), GBP1 is highly likely to possess broad-spectrum anti-coronavirus activity.

As a single-stranded, positive-sense RNA virus, PDCoV stimulates type I and type III interferon production in ST cells in a manner proportional to the viral dose (Fig. 1A through C). GBP1 is an IFN-inducible guanosine triphosphatase (GTPase) gene and is induced by interferon (52). Our study additionally showed that PDCoV triggers the expression of sGBP1 in a viral dose-dependent manner (Fig. 1D and G). Moreover, the sGBP1 mRNA expression is also significantly increased in the PDCoV-infected pigs’ small intestine (Fig. 1H). The findings suggest a correlation between sGBP1 expression levels and the intensity of PDCoV infection. High expression usually indicates a stronger antiviral response.

The mouse GBP1 and human GBP1 have been found to have the antiviral effect against VSV, EMCV, HCV, and KSHV infection (24, 27, 28, 30). The sGBP1 has also been reported to inhibit CSFV, JEV, PRRSV, and PRV (32, 40, 41, 49). Our study first comprehensively proved that the sGBP1 is a key host gene to impress PDCoV replication through over-expression plasmid, stable expression cell strain, and knockout cell strain. The antiviral activity against PDCoV infection involves both the extensive GTPase domain and a C-terminal α-helical segment of sGBP1, which indicates sGBP1 may act through several distinct mechanisms to suppress PDCoV replication (Fig. 4A through D). The hallmark function of GBPs is their GTPase activity, which involves binding to GTP and catalyzing its hydrolysis into GDP. The GTPase activity of GBP1 was demonstrated to be responsible for its antiviral in Flaviviridae virus (HCV, CSFV, JEV) (30, 32, 49), Herpesviridae virus (PRRSV), and Herpesviridae virus (KSHV, PRV) (28, 40, 41). Consistent with the above findings, our research shows that the inhibition of PDCoV replication by sGBP1 also primarily depends on its GTPase activity (Fig. 4). The nucleotide-dependent oligomerization (R224/225) is crucial for the suppression of HCV proliferation (53). But the constitutive homodimerization mutant of sGBP1 does not enhance its antivirus activity, suggesting that the anti-PDCoV activity of sGBP1 might be performed by its monomer (Fig. 4F through H). In JEV infection, the isoprenylation of sGBP1 reduces the expression and cleavage of the prM protein, thereby decreasing the yield of infectious virions (49). The isoprenylation modification is a key post-translational modification of GBP1. The nucleotide-dependent oligomerization (R224/225) and the isoprenylation (C587) also have an antivirus effect on PDCoV (Fig. 4F through H). The constitutive homodimerization mutant sites of GBP1 are related to the affinity of GBP1 to the cell membrane (47, 54), and the isoprenylation modification related to its localization to the Golgi (48). The hGBP1 self-assembly and nucleotide hydrolysis mutant protein changes the subcellular localization of hGBP1 from the cytosol to the plasma membrane (47). This change may affect the operation of the sGBP1 antiviral mechanism.

The IP-MS was utilized to investigate protein interactions occurring throughout PDCoV infection. The protein coverage of NSP8 and NSP9 ranked first and second, respectively (Fig. 5A and B). In our study, we have shown that sGBP1 directly interacts with NSP8 through Co-IP, GST pull-down assay, and confocal microscopy. NSP8 is an essential protein of the coronavirus’ RTC, which is the most important complex governing RNA transcription and replication in coronavirus. The RTC structure of SARS-CoV-2 comprises several viral proteins such as NSP12, NSP7, NSP8, NSP9, NSP13, and RNA (14, 55). NSP8 plays a vital role within the RTC by acting as a scaffold for the binding of NSP7 and NSP12, as well as facilitating the recruitment of the helicase NSP13 to the complex (17). The interaction between NSP8 and sGBP1 suggested that sGBP1 might regulate the conformation of PDCoV RTC. The interaction domain between PDCoV NSP8 and GBP1, PDCoV NSP8 and NSP12, shows that both sGBP1 and NSP12 interacted with the NTD domain of NSP8 (Fig. 6C through E and 7B and C). The competitive Co-IP experiment with sGBP1, NSP8, and NSP12 demonstrated that sGBP1 inhibits the binding of NSP8 and NSP12 (Fig. 7D). Type II interferon suppresses murine norovirus (MNV) replication specifically during the formation of the replication complex (RC). Studies have shown that hGBP1 alone can mediate this IFNG-driven inhibition of MNV replication (56, 57). Furthermore, hGBP1 is directed to the MNV replication complex through the LC3 conjugation pathway (57, 58). These studies found GBP1 target virus RC, but no evidence to indicate that GBPs directly rupture the viral RC. In our study, we first demonstrated that sGBP1 inhibited the RTC formation by competing with the RTC component protein, revealing a novel antiviral mechanism of GBP1.

The NTD domain of SARS-CoV-2 NSP8 protein functions as an anchor, facilitating binding not only to NSP12 but also directly to viral RNA (17). Like SARS-CoV-2 NSP8, the PDCoV NSP8 interacts with Nsp12 and binds with PDCoV RNA (Fig. 7A and 8B). Although NSP12 could not bind to PDCoV RNA, the interaction between NSP12 and NSP8 increases the amount of NSP8 binding to RNA (Fig. 8D). The molecular docking results (a more negative docking score signifies stronger binding) also supported this result. The docking score of NSP8-RNA is −257.71, whereas the NSP8-NSP12-RNA’s docking score is −340.11 (Fig. 8G). This RNA-binding ability increase might be attributed to the interaction between NSP12 and NSP8, which led to a change in the affinity of NSP8 for viral RNA. What’s more, we first found that sGBP1 was able to bind with viral RNA, and co-expression of NSP8 and sGBP1, both of them the RNA binding ability significantly enhanced (Fig. 8E and F). Suggesting that the interaction between NSP8 and sGBP1 might be suitable for binding viral RNA, but it requires in-depth structural studies in the future to confirm our initial observations. However, once sGBP1, NSP8, and NSP12 were co-expressed, the binding amount of NSP8 to RNA significantly decreased. In our study, the AI-based analysis utilizing AlphaFold 3 and HDOCK provided a preliminary explanation for the differential binding affinity of various NSP8 complexes to RNA. The docking score results show that sGBP1 exhibits higher affinity for RNA than NSP8. Furthermore, the binding affinity of NSP8-NSP12-RNA is stronger than NSP8-RNA, and the binding affinity of sGBP1-NSP8-RNA is comparable to that of NSP8-NSP12-RNA (Fig. 8G). These molecular docking results are consistent with the RNA-pull-down findings (Fig. 8F). The above data indicate that the impact of sGBP1 on the RNA-binding ability of NSP8 and the NSP8-NSP12 complex is twofold: First, sGBP1 competes with NSP12 for binding to the NTD domain of NSP8, thereby inhibiting the formation of the functional RTC. Second, sGBP1 alone, or the complex formed by the interaction between sGBP1 and NSP8, possesses a strong RNA-binding potential, which allows it to compete with the RTC for viral RNA. This competition ultimately suppresses the binding of the RTC to RNA and downregulates the transcription and replication of the viral genome. Structure investigation is required to confirm whether the spatial conformation of the RTC undergoes such a change in the future. Our research demonstrates that sGBP1 binds to the genomic RNA of PDCOV to inhibit the formation of the viral RTC complex, thereby downregulating viral replication. However, during coronavirus replication, a series of nested positive-sense and negative-sense subgenomic RNAs are produced, which may possess distinct functional and binding properties. SARS-CoV-2 research revealed that antiviral factors ZC3HAV1, MOV10, EIF2AK2, and TRIM28 interact with its 5′ UTR through MS2 affinity purification coupled with liquid chromatography-mass spectrometry (59). Further investigation is warranted to determine whether sGBP1 binds to PDCoV subgenomic RNAs (sgRNAs) during replication and to identify the exact sequences involved.

In summary, in this study, we first proved that sGBP1 is induced to inhibit PDCoV infection. The sGBP1 exhibits certain antiviral effects through its GTPase activity, homodimerization, and isoprenylation activities, with GTPase activity being predominant. In addition, sGBP1 impairs PDCoV RTC formation by competing with the viral RdRp (NSP12) for binding to the scaffold protein NSP8 of RTC and inhibits RTC binding with virus RNA. This research uncovered a previously unrecognized antiviral function of GBP1, offering a promising target for strategies aimed at preventing and managing coronavirus infections.

## MATERIALS AND METHODS

### Cells

ST and HEK-293T cells were maintained in Dulbecco’s modified Eagle’s medium (DMEM) (Thermo Fisher, USA) supplemented with 10% fetal bovine serum (FBS) (Gibco, USA), along with 100 U/mL penicillin and 100 mg/mL streptomycin, incubated at 37℃ in an atmosphere containing 5% CO_2_. ST-sGBP1 stable overexpressing cell lines were generated by the lentivirus system as previously described (60). The ST-*Gbp1* knockout cell was generated using CRISPR/Cas9. The sgRNA sequence is as follows: sGBP1-sgRNA forward, ATCGTGGGCCTGTACCGCACAGG, and reverse, CCTGTGCGGTACAGGCCCACGAT. The edited monoclonal ST cells underwent a sequencing and western blot assay, and the correct cell line was named ST-*sGbp1^−/−^*.

### Viral infection

The PDCoV HeN17 strain (GenBank accession no. OR230676.1) was employed and referred to as “PDCoV” throughout this study. Wild-type ST cells and sGBP1-overexpressed ST cells were infected with PDCoV at the specified MOI for 2 h. Following incubation in DMEM supplemented with 10 μg/mL trypsin. For PDCoV infection in *vivo*, 5-day-old pigs were divided into two groups: the challenge group (*n* = 5) was orally inoculated with 10^5^ TCID_50_ of the HeN17 strain. In contrast, the control group (*n* = 5) received an equivalent volume of DMEM. All piglets were sacrificed at 48 h post-infection, and intestinal tissues were collected.

### Plasmids, antibodies, and reagents

PCAGGS-FLAG, pCAGGS-HA, and pCAGGS-MYC empty plasmids were kindly provided by the Changjiang Weng lab. The swine Gbp1 (sGbp1), sGbp1_1-315_, sGbp1_316-590_, sGbp1_K51A,_ sGbp1_R224/225E,_ sGbp1_C587A,_ PDCoV Nsp8, and Nsp12 were cloned into the pCAGGS plasmid and used to transfect ST cells or HEK293T cells.

The monoclonal antibodies (MAb) against the FLAG tag, Myc tag, and HA tag were purchased from Cell Signalling Technology (CST, Massachusetts, USA). The anti-GBP1 (15303-1-AP (R), 67161-1-Ig (M)) and anti-actin (20536-1-AP) were purchased from Proteintech (Wuhan, China). The polyclonal antibodies (pAb) against the N protein and NSP8 protein of PDCoV were prepared in our laboratory.

### qPCR

At the specified time points, cells were collected, and total RNA was extracted employing the MiniBEST Universal RNA Extraction Kit (TaKaRa). And then, transcribe the RNA into cDNA by HiScript IV All-in-One Ultra RT SuperMix for qPCR (Vazyme, China). The PDCoV copies were detected by the M gene Taqman probe (AceQ Universal U+ Probe Master Mix V2, Vazyme, China). The PDCoV N gene sgRNA, genomic RNA (ORF1a/1b sgRNA), sGbp1, and gapdh genes mRNA expression were detected by SYBR GreenIQPCR detecting assay (Taq Pro Universal SYBR qPCR Master Mix, Vazyme, China). The qPCR detected primers or probe as following: PDCoV-M-F: 5′-ATCGACCACATGGCTCCAA-3′, PDCoV-M-R: 5′-CAGCTCTTGCCCATGTAGCTT-3′, PDCoV-M-probe: 5′-FAM-CACACCAGTCGTTAAGCATGGCAAGCT-BHQ-3′; PDCoV-N-F: 5′-AGCAACCACTCGTGTTACTTG-3′, PDCoV-N-R: 5′-CAACTCTGAAACCTTGAGCTG-3′; PDCoV-ORF1a/1b-F: 5′-CTAGTGCCTGTATAGACGCTGGTC-3′, PDCoV-ORF1a/1b-R: 5′-GCCATCACTATCCATACACTCACAC-3′; sGbp1-F: 5′-GAAGGGTGACAACCAGAACGAC-3′, sGbp1-R: 5′-AGGTTCCGACTTTGCCCTGATT-3′; Gapdh-F: 5′-CCTTCCGTGTCCCTACTGCCAAC-3′, Gapdh-R: 5′-GACGCCTGCTTCACCACCTTCT-3′.

### Co-IP and western blot assay

Co-IP and western blotting were conducted following established protocols (60). The resulting lysates were separated into input and IP fractions. For the input samples, lysates were mixed with 1× loading buffer and heated at 100°C for 10 min. The IP portion of cell lysates was incubated with Anti-DYKDDDDK Affinity Resin Easy for 1 h on a roller at room temperature. After incubation, centrifuge the tube for 1 min at 1,000 *g* and wash the precipitated beads with PBST 5 times. For endogenous Co-IP, the cell lysates were incubated with rabbit IgG and rabbit polyclonal antibody against NSP8, respectively, at 4°C overnight on a roller. And then, added protein A+G beads into the lysates and incubated for 1 h on a roller at room temperature. PBS was used to wash the incubated beads five times. After washing, the beads were added to 2 × loading buffer and boiled for 10 min. The samples were first resolved by 10%–12% SDS-PAGE and then transferred onto a PVDF membrane (Millipore) for western blotting. The membranes were blocked for one hour using 5% BSA in PBST. After washing three times with PBST, they were incubated consecutively with primary antibodies, followed by secondary antibodies. The detection of the western blot signals was performed by a chemiluminescence imaging system (Fine-do X6; Tanon, China).

### Immunofluorescence and confocal microscopy

To observe PDCoV infection, cells were cultured on dishes suitable for confocal microscopy and separated into mock and infected groups. The infected cells were treated with PDCoV at an MOI of 0.1 for 12 h. After infection, cells were fixed in 4% paraformaldehyde for 30 min and washed once with PBS. Permeabilization was carried out using 0.3% Triton X-100 in PBS for 20 min, followed by blocking with 5% BSA in PBS for 1 h. The cells were subsequently incubated with antibodies specific to the PDCoV N protein for 1 h and then stained with Alexa Fluor 488-labeled goat anti-rabbit IgG for another hour. Finally, nuclear DNA was counterstained using DAPI. The green fluorescence indicating PDCoV N protein presence was visualized and documented with a fluorescence microscope (Carl Zeiss AG, Oberkochen, Germany).

To examine the subcellular localization of HA-sGBP1 and FLAG-NSP8, HEK293T cells were cultured on confocal dishes and transfected with plasmids expressing either FLAG-NSP8, HA-sGBP1, or both for 24 h. Subsequently, cells were processed following the immunofluorescence assay (IFA) protocol, which included fixation, membrane permeabilization, blocking, incubation with primary antibodies, secondary antibody staining, and DAPI nuclear staining prior to imaging. The HA tag was detected using a rabbit-derived primary antibody, followed by an Alexa Fluor 488-conjugated goat anti-rabbit IgG secondary antibody. For the FLAG tag, a mouse-derived primary antibody was applied, with Alexa Fluor 633-labeled goat anti-mouse IgG serving as the secondary antibody. Visualization of HA-sGBP1 and FLAG-NSP8 localization was performed using a Zeiss LSM-710 laser scanning confocal microscope (Carl Zeiss AG, Oberkochen, Germany) equipped with a 63× oil immersion objective.

### GST pull-down assay

GST-NSP8 and GST control proteins were obtained via affinity chromatography with glutathione-Sepharose 4B (GE Healthcare Life Sciences) and eluted with 10 mM reduced glutathione. To assess protein interactions, 10 μg of purified GST-NSP8 or GST was incubated with an equivalent amount of His-sGBP1. The assembled protein complexes were then purified utilizing glutathione-Sepharose 4B beads and analyzed by immunoblotting with anti-GST and anti-His antibodies.

### RNA pull-down assay

The RNA pull-down assay was performed according to a previously described method (61). HEK293T cells were transfected or co-transfected with plasmids expressing HA or FLAG-tagged sGBP1, HA-tagged NSP12, and MYC-tagged NSP8 as indicated. Cells were lysed using a buffer containing 1× protease inhibitor (Thermo). A total of 20 μg of PDCoV HeN17 RNA was either left unlabeled or labeled with 20 μg photobiotin under strong light exposure (450 W, 220 V). Both unlabeled and biotinylated RNAs were incubated with equal amounts of HEK293T cell lysates overexpressing GBP1, NSP8, or NSP12, in the presence or absence of unlabeled PDCoV RNA, within RIP buffer supplemented with 100 U/mL RNase inhibitor RNaseOUT (Invitrogen) and 1× protease inhibitors. The mixture was gently rotated at 4°C for 4 to 8 h. Afterward, biotinylated RNA-protein complexes were isolated using Dynabeads M-280 streptavidin (Invitrogen) on a magnetic rack. The precipitated complexes were washed five times with ice-cold washing buffer, resolved by 12% SDS-PAGE, and analyzed by Western blotting.

### Structure predictions and molecular docking

The AlphaFold 3 online server (https://alphafoldserver.com/) was employed to predict protein complex models, and Hdock was used to calculate docking scores between protein complexes and RNA according to published methods (62–64). The predicted template modeling (pTM) score and the interface pTM (ipTM) score were used to assess the accuracy and the confidence in interchain interactions of the prediction model, respectively. For the protein-RNA complex model, the dsRNA sequence of the 5′ UTR of PDCoV was used. The RNA sequence is 5′-ACUAGUCACUAGGUGUAAGUGAUCUGAUCUGGGCGUAUUGUGUUGCGC-3′. Following modeling, HDOCK was employed to calculate the binding potential between the protein complex structure and the RNA. The resulting model files were first converted to PDB format and submitted to the HDOCK platform. Using HDOCKlite, a fast Fourier transform (FFT)-based rotational and translational search was performed to screen for conformations with high shape complementarity. Candidate conformations were then iteratively evaluated using the ITScore family of functions, and a final composite score was calculated by integrating multiple energy terms. All poses were ranked in ascending order of their Docking Score (from negative to positive), with the top-ranked pose (Top 1) representing the optimal predicted conformation. The Docking Score is inversely correlated with predicted binding stability and affinity; a more negative value signifies stronger binding.

### Statistical analysis

Statistical evaluation was performed utilizing unpaired and two-tailed Student’s *t*-tests, as well as one-way or two-way ANOVA, with Bonferroni’s post hoc correction applied subsequently. Findings were considered statistically significant when *P* values were less than 0.05.

## Data Availability

The PDCoV HeN17 strain complete genome (GenBank: OR230676.1) is available on https://www.ncbi.nlm.nih.gov/nuccore/OR230676.1/. The AlphaFold 3 online server website is https://alphafoldserver.com/. The HDOCK web server is available at http://hdock.phys.hust.edu.cn/. The data are available from the corresponding author on reasonable request.
